# A conceptual framework of stress vulnerability, depression, and health outcomes in women: potential uses in research on complementary therapies for depression

**DOI:** 10.1002/brb3.249

**Published:** 2014-07-10

**Authors:** Patricia A Kinser, Debra E Lyon

**Affiliations:** 1 Department of Family and Community Health Nursing, School of Nursing, Virginia Commonwealth UniversityPO Box 980567, Richmond, Virginia, 23298-0567; 2 School of Nursing, University of FloridaPO Box 100197, Gainesville, Florida, 32610-0197

**Keywords:** Biopsychosocial resource, complementary therapy research, conceptual framework, depression, stress, women, yoga

## Abstract

**Background:**

Depression is a chronic mental health condition that affects millions of individuals worldwide. It is well-established that psychological stress plays an integral role in depression and that depression has numerous negative health outcomes. However, a closer look at components of stress vulnerabilities and depression is required to allow for the development and testing of appropriate interventions.

**Aims and Discussion:**

This article describes a conceptual framework about the complex and bidirectional relationship between stress vulnerability, depression, and health outcomes in women. The authors elucidate how the framework can be applied in clinical research about cellular aging and on the mechanisms of complementary and alternative medicine (CAM) for depression, using yoga as an example of a CAM modality.

**Conclusion:**

The proposed conceptual framework may be helpful for adding depth to the body of knowledge about the use of mind-body therapies for individuals at high risk of stress vulnerability and/or depression.

## Introduction

Depression is a leading cause of disability and disease burden worldwide and in the United States, affecting millions of individuals worldwide, particularly women. Women have a high risk of experiencing depression with an estimated lifetime risk of 10–25% (Kessler et al. 2003; Shenal et al. 2003). This increased vulnerability to depression starts in puberty and continues through menopause (Nolen-Hoeksema 2006; Deecher et al. 2008). Depression is of public health concern because of the short and long-term detrimental effects to the woman and her family. Individuals with depression experience high rates of anxiety, suicidality, substance use, and poor spouse/child relations (Kessler et al. 2008; Sunderland et al. 2010; Zbozinek et al. 2012); depression is also highly related to prevalent health outcomes such as cardiovascular disease, one of the five major causes of death in the United States (Minino and Murphy 2012; Elderon and Whooley 2013). The core symptoms of a major depressive episode are: a persistent depressed mood, difficulty concentrating or decision making, decreased energy, loss of interest in previously pleasurable activities, weight changes, changes in sleep (insomnia or hypersomnia), psychomotor changes (agitation or retardation), a pessimistic outlook with or without suicidal ideation (American Psychiatric Association 2013).

Appropriate treatment of depression is essential, yet many depressed women find the usual depression care (e.g. antidepressant medications and/or psychotherapy) to be inappropriate due to various concerns about cost, side effects, or inadequate relief of symptoms (Schreiber and Hartrick 2002; Lafrance and Stoppard 2006; Romans et al. 2009). Many individuals with depression experience persistent depressive symptoms despite the usual depression care (Zajecka et al. 2013), prompting them to seek additional relief through adjunctive or complementary therapies (Jorm et al. 2002, 2008). For example, mind-body therapies, such as yoga, have received attention in both the lay and research literature as possible adjunctive therapies for depression (Bussing et al. 2012; Cramer et al. 2013). In order to thoroughly examine these and other similar interventions, research should be guided by a conceptual framework which incorporates and evaluates the relationship of individual, social/environmental, biological, and psychobehavioral factors involved in depression and the impact of the intervention on these factors. Although research is promising about nonpharmacologic complementary therapies such as yoga for depression, herein we suggest that use of the proposed conceptual framework may be helpful for adding depth to the body of knowledge about the use of adjunctive therapies for women with depression.

## A Conceptual Framework of the Relationship between Stress Vulnerability and Depression in Women

Depression and stress have a bidirectional relationship whereby depression may be both a cause and an effect of psychological stress (Kinser et al. 2012). Typically, the brain moderates the effects of stressors to maintain optimal functioning. Microprocesses regulate neurotransmission, endocrine, and immune functioning centrally, and sympathetic and parasympathetic activity in the periphery, all of which maintain allostasis or psychological and physical balance (McEwen and Lasley 2003; Peters and McEwen 2012). In the short term, these regulatory functions enhance the individual's response to stressors and the ability to manage negative physiological effects (Epel 2009). However, when stressors continue unabated, these same processes begin to impair neuronal function and other regulatory systems (Logan and Barksdale 2008; Kinser et al. 2012). The cumulative wear and tear associated with these physiological efforts to manage chronic stressors can cause depression and additional comorbidities. Without the availability and use of biopsychosocial resources, long-term exposure to the chronic stress of depression and/or repeated episodic life stressors can overload one's coping capacity; this may place an individual in a continuous cycle of stress response with negative affect states which can decrease quality of life and increase morbidity and mortality (McEwen 2000, 2007; McEwen and Lasley 2003; Luyten et al. 2006; Clark et al. 2007; Taylor et al. 2010). It has been suggested that high levels of stress and depression are associated with accelerated cellular aging, a potential biomarker of the overloaded coping capacity of an individual (Kinser and Lyon 2013).

## Stress Vulnerability: Individual Chronic/Acute Burdens, Biological and Psychosocial Environment

**Figure 1:**
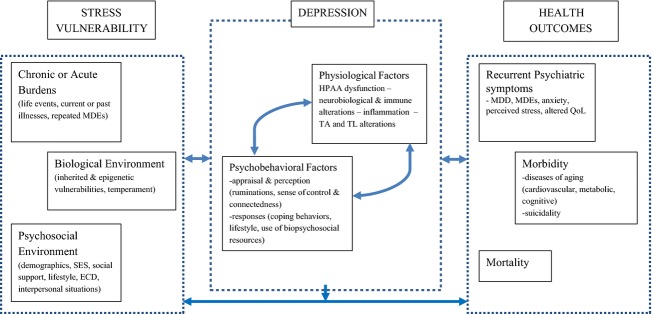
Conceptual framework of individual stress vulnerability, depression, and health outcomes in women. SES= socioeconomic status; ECD= early childhood development; HPAA= hypothalamus-pituitary-adrenal axis; TA= telomerase activity; TL= telomere length; MDD= major depressive disorder; MDEs= major depressive episode; QoL= quality of life.

The cyclic interplay of stress vulnerability, depression, and health outcomes is represented in the conceptual framework shown in Figure 1. The framework represents how stress vulnerabilities play an important role in depression and health outcomes in women (Kinser and Lyon 2013). Stress vulnerabilities are based upon numerous factors associated with acute and chronic stress, including *individual chronic/acute burdens*, the *biological environment*, and the *psychosocial environment*. The variety of complex and potential stressors in an individual's life may interact and contribute to increased risk of depression. In addition, the experience of depression may heighten an individual's tendency toward experiencing stressful episodes. Persistent and profound stressors may prevent regulatory mechanisms from adjusting appropriately, continuing the cycle of neurobiological dysregulations, poor health outcomes, and potentially advanced cellular aging (Nolen-Hoeksema 2006; Kinser et al. 2012; Kinser and Lyon 2013).

*Individual chronic and acute burdens* involve an accumulation of life stressors that may include current or past stressful life events and current or past illnesses (e.g., chronic or acute psychological or physical illnesses). Acute stressful life events and current or past illnesses may precipitate or exacerbate depressive symptoms. For example, rodent studies and preliminary human studies suggest that the quality of the early childhood environment can shape brain development with associated changes to neuroanatomical structure/function and receptor levels/gene expression (Curley et al. 2011); theoretically, any of these changes may either be adaptive (and lead to adaptive behaviors and decreased risk of depression) or disruptive (and lead to unhealthy behaviors and a high risk for depression) (Garner et al. 2012). Stressful early childhood experiences can significantly undermine the development of adaptive coping skills required to deal with challenges in adulthood and may also create the foundation for unhealthy lifestyles, negative interpersonal relationship patterns, and poor health outcomes (Garner et al. 2012; Danese and McEwen 2012; Shonkoff et al. 2012). As another example, studies suggest that women with a history of childhood traumas, such as sexual abuse, and low levels of current social support are at higher risk of unintended pregnancies, which are associated with prenatal and postpartum depression (Mercier et al. 2013; Nelson and Lepore 2013). To continue this example, a woman with an unintended pregnancy may find herself unprepared to serve in a social role in which she is expected to put others' needs ahead of her own, which can be acutely and chronically stressful and is highly related to both prenatal and postpartum depression (Mercier et al. 2013).

Vulnerabilities regarding the *psychosocial environment* that play a role in the impact of depression on health outcomes may be demographics/socioeconomic status, perceived social support, lifestyle, and interpersonal situations. Socioeconomic status is clearly linked to stress vulnerability, as seen in low-income populations which have high levels of stress, impaired coping, and depression. Persistent socioeconomic inequalities are linked with stress vulnerability, particularly with regards to educational and financial opportunities; these inequalities are also linked with health disparities and unhealthy lifestyle choices and poor health outcomes (Shonkoff et al. 2009; Danese and McEwen 2012). Negative interpersonal situations, such as intimate partner violence (IPV) and low perceived social support, add additional vulnerability for stress and depression in women (Devries et al. 2013); fortunately, long-term sequelae of traumatic experiences such as IPV and early childhood abuse may be attenuated by higher perceived social support (Kaufman et al. 2004; Chuang et al. 2012). Nonviolent gender-specific interpersonal situations may also place additional wear and tear on women's mental and physical health, particularly if there are feelings of guilt or selfishness associated with participating in healthy self-care activities rather than focusing on the financial or other needs of the family (Hauenstein and Boyd 1994; Lafrance and Stoppard 2006; Hauenstein and Peddada 2007; Hauenstein et al. 2007; Petterson et al. 2009). There also appear to be *biological vulnerabilities* that affect women, in particular, such as temperament, inherited and epigenetic changes and dysregulated stress response systems (Nolen-Hoeksema and Girgus 1994; Nolen-Hoeksema et al. 1999; Davidson et al. 2002; Hammen 2005; Nolen-Hoeksema 2006; Halbreich and Kahn 2007; Deecher et al. 2008; Gotlib and Hammen 2009; Taylor et al. 2010; Young and Korszun 2010). Epigenetic research suggests that social experiences in both childhood and adulthood may significantly modulate stress reactivity and depression via a multitude of mechanisms (for a more thorough review, see (Curley et al. 2011).

## Depression: Psychobehavioral and Physiological Factors

*Psychobehavioral factors* play an important role in the development and maintenance of depressive states. A woman's perception of stress and her response to that appraisal are important moderators in the relationship between stress and depression. With regard to stress perception, a key individual factor related to the effect of stress is the degree to which an individual perceives that stress to be significant and to what degree the individual thinks she, in this case, has control over the situation (Lazarus and Folkman 1984). Important factors of stress perception are related to individual differences in personality and cognitive styles in the face of stressful life situations; these may either increase or decrease an individual's risk for and experience of depressive illness. For example, two key aspects of these individual styles are relevant to the bidirectional relationship between stress vulnerability and depression. First, every individual has their own sense of control in the face of stress and depression. Extensive cross sectional and longitudinal research has provided evidence to suggest that individuals with stressful life situations, because of environmental factors outside of the individuals' control, have higher stress-related psychoneuroimmunologic changes (Hauenstein 1996; Geronimus et al. 2006; Glover et al. 2006; Kahn and Pearlin 2006; Clark et al. 2007; Johansson et al. 2007). Second, every individual has more or less tendency toward ruminations, or persistent repetitive negative thinking. Ruminative patterns may be normative because, evolutionarily speaking, humans must pay close attention to stressors or interpersonal distress to maintain safety and social relations (Buss 2000; Seligman et al. 2006). However, in modern society, those with heightened attention to and perception of stressors may, in fact, have a biased perception toward negative emotions; for example, increased levels of ruminations on stress may affect levels of depression (Seligman et al. 2006). When experiencing depression, women quite often report negative ruminations, which are in-turn related to low self-esteem, hypersomnia, and anxiety (Kendler et al. 2002; Keita 2007; Marcus et al. 2008; Rochlen et al. 2010). Ruminations may be particularly problematic for women because they can increase the stress of depression by inducing negative thoughts about the past, present, and/or future. Ruminations are associated with lower levels of social support and increased suicidal ideations, all of which continue the cycle of stress and prolonged depression (Nolen-Hoeksema et al. 2008).

Another key *psychobehavioral factor* in depression is whether an individual has personal and environmental resources for appropriate stress management. The ability to respond to stress and depression in a healthy manner is highly dependent on the availability and use of biopsychosocial resources. To maintain health, an individual must be aware and capable of/interested in using relevant resources. In particular, the use of healthy biopsychosocial resources is a protective mechanism essential for the capacity of women to deal with stressors; in essence, the ability to rapidly reach a sense of equilibrium or return to a calm baseline can be highly protective (Danner et al. 2001). Research suggests that those with depression and anxiety disorders tend to pick ineffective health maintenance strategies, whereas happier individuals have tendencies to reach out for social support and healthy biopsychosocial strategies and are more willing to use positive appraisal techniques about life stressors (Diener et al. 2006). Despite these tendencies, however, interventions may be effective for assisting individuals in their use of healthy, positive biopsychosocial resources. Biopsychosocial resources may include a wide range of activities and behaviors. For example, positive health behaviors involving healthy nutrition, exercise, relaxation, and healthy sleep patterns may help women to effectively respond to acute or chronic stressors in their lives (Romans et al. 2009; Hauenstein 1996; Chang et al. 2011; Dusek et al. 2008; Institute of Medicine (US) Committee on Sleep Medicine and Research et al. 2006; Ruiz-Nunez et al. 2013; Clark et al. 2011; Sims et al. 2008; Dunn et al. 2005). Mental wellness may be promoted by psychological therapies that emphasize positive emotions, gratitude, personal strengths, and engagement with life (Seligman et al. 2006; Emmons 2008). Furthermore, a significant factor in stress moderation may be the participation in intentional activity, or discrete actions that require behavioral and/or cognitive and/or volitional effort (Lyubomirsky et al. 2005). Intentional activities, particularly those that focus on personal strengths, positive emotions, and mindfulness, may stimulate a healthy stress appraisal and may greatly assist depressed individuals to decrease ruminations that often impact mood (Fredrickson 2008); for example, yoga may be a reasonable intentional activity that meets these needs (Kinser et al. 2012). Finally, an accumulation of experiences through the use of healthy biopsychosocial resources that meet the human needs of competence and social relatedness may allow for mental wellness and decrease the risk of accelerated cellular aging and poor health outcomes (Lyubomirsky et al. 2005).

## Physiological Factors

Extant research suggests that depression is associated with, causes, and/or may be caused by a number of biological perturbations. Support for the interrelationships among biological pathways and depression has been documented via multiple different pathways. Over the past several decades, multiple categories of biological alterations have been associated with depression, including heightened inflammatory activation (Miller et al. 2009), hyperactivity of the hypothalamic-pituitary-adrenal axis (Pariante and Lightman 2008), and more recently, genetic and epigenetic alterations (Massart et al. 2012). Currently, yet another plausible biological link is being explored: the link between the microbiome and depression, focusing on the activation of the central nervous system (CNS) signaling systems by gastrointestinal bacteria (Cryan and Dinan 2012). The accelerated aging hypothesis combines several biological theories to create a theoretically based model of interactions among multiple biological events. Although these mechanisms have been well supported in animal models, human trials are still underway in establishing these mechanisms.

## Health Outcomes

The ability of an individual to respond to the chronic stress associated with depression and to individual life stressors varies greatly depending upon the availability and use of healthy biopsychosocial resources (Hauenstein 1996; Kiecolt-Glaser et al. 2002; McCain et al. 2005; Uebelacker et al. 2010a). *Poor health outcomes* may occur if these resources are not available or used; psychological and physical health may decline and lead to decreased psychosocial functioning, decreased health-related quality of life, and increased incidence of comorbid conditions (McEwen and Lasley 2003; Luyten et al. 2006; Clark et al. 2007; McEwen 2007; Taylor et al. 2010). There is accumulating research on the relationship of depression with morphologic changes including decline in gray matter density of the hippocampus, anterior cingulum, left amygdala, and right dorsomedial prefrontal cortex (Frodl et al. 2008) and hippocampal volume loss (McEwen 2005). In addition to brain-specific alterations, there is accumulating research on the adverse effects of depression on comorbid conditions. The adverse health outcomes of untreated major depression are decrements in health that surpass the effects of chronic diseases angina, arthritis, asthma, and diabetes (Moussavi et al. 2007). Individuals with depression have higher rates of obesity, cardiac conditions including hypertension, heart disease, and diabetes than the general population (Katon 2008, 2011; Clarke and Currie 2009; Pozuelo et al. 2009).

## Using the Conceptual Framework: Potential for Complementary and Alternative Medicine (CAM) Research

This framework may be useful when considering research studies on CAM for individuals with depression. The intent of the remainder of this article is to discuss yoga as an example of a CAM modality and how this intervention could influence various aspects of the conceptual framework of the complex relationship of stress and depression in women. In order to reduce risk of poor health outcomes, complementary therapies such as yoga may provide a stress-buffering effect to enhance health and relieve effects of chronic stress and depression (Loizzo 2009). However, stressful experiences and negative ruminations may contribute to the cycle of stress and depression, yoga may allow for a return to balance of the multiple components involved in mental wellness. Yoga may be an effective biopsychosocial intervention for dealing with the cycle of stress and depression because yoga involves components designed to have an effect on key aspects of depression in women, from physical activity to meditative, relaxing practices to rhythmic, soothing breathing practices to social interactions (Dunn et al. 2001, 2005; Weintraub 2004; Netz et al. 2005; Larun et al. 2006; Marcus et al. 2008; Tsang et al. 2008; Saeed et al. 2010; Uebelacker et al. 2010a,b). Yoga philosophy emphasizes the use of yoga for mental and physical wellness personalized to the individual's needs (Kinser and Williams 2008), and this may appeal to women who are uncomfortable with interventions based solely on the biomedical model that focuses primarily on neurochemistry. Studies have shown that women often attribute their experiences of and recovery from depression to life experiences and social factors rather than to biochemical pathology or medications (Schreiber and Hartrick 2002; Lafrance and Stoppard 2006).

The proposed conceptual framework is a natural complement to the biomedical model that is commonly used in western societies because it maintains an equal focus on biology and the particular needs/experiences of women. Furthermore, many researchers suggest that women with depression seek out complementary therapies because the usual allopathic pharmacologic care does not adequately address their individual symptoms or explanatory model of depression or may have unappealing side effects and expense (Hammen 1992, 2005; Schreiber and Hartrick 2002; Kessler et al. 2003; Pirraglia et al. 2004; Saper et al. 2004; Kirkwood et al. 2005; Lafrance and Stoppard 2006; Gotlib and Hammen 2009; Uebelacker et al. 2010a). Therefore, a need exists to investigate the effects of complementary interventions in women who have depression using this integrative framework that acknowledges the complex relationship between depression and stress in women.

The conceptual framework is relevant for research studies testing the use of complementary therapies for women with depression because it provides moderating and outcome variables for measurement. In particular, the framework suggests a number of potential moderating variables: *stress perception* (individual life stressors, ruminations) and *responses* (use of healthy biopsychosocial resources). The framework provides a number of outcome variables for measurement consistent with the literature on complementary therapies for depression, stress, ruminations, and anxiety. This framework is also suggested for use in future research on yoga because it provides an obvious target for intervention: an individual's response to stress and depression through the availability and use of biopsychosocial resources. Interventions, like yoga, for treating depression are needed that address gender-specific issues and symptoms, and empower women to participate in positive health promoting self-care activities (Schreiber and Hartrick 2002; Hammen 2005; Lafrance and Stoppard 2006; Nolen-Hoeksema 2006; Nolen-Hoeksema et al. 2008; Gotlib and Hammen 2009; Nolen-Hoeksema and Hilt 2009). However, women with depression may have difficulty sustaining behaviors for personal health and wellness, which highlights the importance of conducting research based upon the proposed conceptual framework (Lafrance and Stoppard 2006; Gotlib and Hammen 2009).

It has been theorized that the practice of yoga as a healthy biopsychosocial resource may assist individuals with depression to cope with stress and thus enhance their mood. Studies suggest that individuals participating in various yoga interventions report decreases in psychological and physical symptoms of depression and stress (Uebelacker et al. 2010a). Cramer and colleagues (2013) have recently conducted a meta-analysis of studies on yoga for depression, finding that yoga may be effective for short-term remission of depression (Cramer et al. 2013). Numerous putative mechanisms have been suggested to explain the beneficial effects of yoga, including those which enhance global regulation of stress response systems (Kinser et al. 2012). For example, Streeter and colleagues (2012) suggest that yoga corrects underactivity of the inhibitory neurotransmitter gamma amino-butyric acid [GABA] with resultant decreases in depression symptoms (Streeter et al. 2010, 2012). As another example, emerging evidence suggests that depression is related to alterations in biological markers, such as inflammatory cytokines and related DNA methylation patterns, which might influence mental health outcomes.(Uddin et al. 2011, 2013; Akbarian and Nestler 2013; Penninx et al. 2013) It appears that the impact of depression-related physiologic changes are potentially reversible with interventions such as yoga (Schmidt et al. 2013; Yehuda et al. 2013). For example, remarkable findings from a recent study suggest that the therapeutic potential of interventions involving mindfulness may stem partially from the epigenetic control of inflammatory processes (Kaliman et al. 2014). Yogic practices that enhance stress regulation by inducing relaxation appear also to induce epigenetic changes in the expression of proteins involved in energy metabolism, mitochondrial function, insulin secretion, telomere maintenance, and inflammation (Bhasin et al. 2013). Despite these interesting findings, a complication in the development of evidence-based recommendations about yoga for depression in women is that many yoga research studies have methodological limitations and do not integrate an evaluation of individual, social/environmental, and physiological factors in their evaluation of outcomes. Using the proposed conceptual framework that is not only specific to the population of interest (women with depression) but also includes relevant variables for measurement may allow researchers to add substantively to the body of knowledge about yoga as an effective complementary therapy.

## Conclusion

The model in Figure 1 elucidates the bidirectional relationship of stress vulnerabilities, depression, and health outcomes and provides a conceptual framework for the conduct of research about CAM modalities and depression in women. This framework is relevant and timely because it integrates multiple models and theories of the etiology of depression within a context of cellular aging. We suggest that, by providing moderating and outcome variables for measurement, this framework may be helpful for researchers interested in testing the use of complementary therapies, such as yoga, for women with depression.
